# Challenging Autologous Breast Reconstruction in Low BMI Patients with Profunda Artery Perforator (PAP) Flap: Impact of Skin Island Design on Complication Rates and Long-Term Aesthetic Outcomes

**DOI:** 10.3390/jcm14113707

**Published:** 2025-05-25

**Authors:** Selina Neurauter, Maria E. Casari, Angela Augustin, Theresia Stigger, Christine Brunner, Dolores Wolfram

**Affiliations:** 1 Department of Plastic, Reconstructive and Aesthetic Surgery, Medical University of Innsbruck, Anichstrasse 35, 6020 Innsbruck, Austria; selina.neurauter@tirol-kliniken.at (S.N.);; 2 Department of Gynecology and Obstetrics, Medical University of Innsbruck, Anichstrasse 35, 6020 Innsbruck, Austria

**Keywords:** breast, breast cancer, autologous breast reconstruction, BREAST-Q, free flap, flap surgery, long term outcome, PAP flap, POSAS, profunda artery perforator flap, vertical PAP flap

## Abstract

**Background:** The Profunda Artery Perforator (PAP) flap is a viable alternative to the Deep Inferior Epigastric Perforator (DIEP) flap, particularly for patients with low BMI and therefore insufficient abdominal tissue. To reduce the high complication rate, especially in our low BMI patient population, we have adapted the use of the vertical skin island design. This study compares complication rates and long-term outcomes of vertical versus horizontal skin island designs in PAP flap breast reconstruction. **Methods:** This prospective, single-center study included 20 patients who underwent PAP flap breast reconstruction. Quality of life and scar quality were assessed using the BREAST-Q and POSAS questionnaires. Additionally, the cosmetic outcomes were analyzed by four plastic surgeons. **Results:** Mean BMI in the vertical group was 23.9 kg/m^2^ and 22.7 kg/m^2^ in the horizontal group. Mean flap weight was 326 g for the vertical group and 355 g for the horizontal group. Fewer complications were observed at the donor site in the vertical group (Clavien–Dindo Classification 3b at donor site: *p* = 0.25). The BREAST-Q evaluation revealed significantly better results regarding the psycho-social well-being (*p* = 0.04) in patients with the horizontalskin island design. Scar evaluation using the POSAS revealed that the scar was perceived as thinner (*p* = 0.02), less pigmented (*p* = 0.03), and showed less relief (*p* = 0.02) in the vertical group. No significant difference was observed in the overall scar assessment by observers (*p* = 0.46). The aesthetic analysis by plastic surgeons showed significantly better results in the horizontal group. **Conclusions:** The vertical skin island design in PAP flap breast reconstruction was associated with lower complication rates and better scar quality compared to the horizontal design. Surgeons, however, rated the overall aesthetic outcome of the vertical design less favorably. These findings highlight the importance of balancing donor site morbidity with overall aesthetic results.

## 1. Introduction

Breast reconstruction following mastectomy is a life-quality enhancing aspect of comprehensive care for breast cancer patients, offering these women an opportunity to regain both physical and psychological well-being [1]. When it comes to autologous breast reconstruction, the Deep Inferior Epigastric Perforator (DIEP) flap has been considered the gold standard for many years [2,3,4]. However, the Profunda Artery Perforator (PAP) flap, introduced by Allen in 2012 [5], has emerged as a promising alternative to the Deep Inferior Epigastric Perforator (DIEP) flap [3,4,6,7,8,9,10]. Specifically for women who lack sufficient donor tissue or for those where abdominal tissue is no longer an option, such as due to scarring from prior surgeries, previous scientific studies have shown that the PAP flap is a valuable alternative [8]. Also, a recent study conducted by our research group demonstrated similar results in terms of donor site morbidity, scar quality, and the aesthetic outcome of the breast comparing the PAP flap and the DIEP flap [11]. Additionally, the PAP flap has not only gained increasing recognition for other indications in recent years, such as lower extremity and head or neck reconstruction, but also for coverage of the ischial or perineal regions [12,13]. In our Department, we offer the PAP flap to slim patients for whom a DIEP flap is not feasible but who still desire autologous tissue reconstruction. As a result, our patient cohort of breast reconstruction with PAP flap tends to be lean with an average BMI of 23.3 kg/m^2^ (vertical skin island group: 23.9 kg/m^2^, range: 19.4–32.4 kg/m^2^, horizontal skin island group: 22.7 kg/m^2^, range: 18.5–27.5 kg/m^2^). In other studies, the BMI of the patients was reported to be higher, ranging from 23.3 kg/m^2^ to 29.1 kg/m^2^ [5,6,14,15]. Early descriptions of the PAP flap involved a horizontal skin island, which raised concerns related to thigh undermining and suboptimal scarring [14,15]. These complications were even more evident in our patient cohort due to the low BMI. Jo et al. [8] also used the PAP flap for slim patients (average BMI = 22.2 kg/m^2^) with 4.2% donor site wound necrosis. Several modifications of the skin island design have been introduced to optimize the donor site complication rates, such as an extended design [16], a fleur-de-lis design [17], or a vertical design [18,19,20]. To address these challenges, we aimed to explore the potential advantages of utilizing a vertical skin island in PAP flap breast reconstruction compared to the horizontal flap island design. To our knowledge, no study has yet compared the aesthetic outcomes and complication rates of the vertical and horizontal skin island in PAP flap breast reconstruction. We believe that the adjusted skin island design positively affects donor site complications. By optimizing this approach, we can enhance breast reconstruction outcomes for challenging low-BMI patients.

## 2. Materials and Methods

### 2.1. Study Design

Approval of this study was obtained by the Institutional Ethics Committee of the Medical University of Innsbruck (protocol code: 1057/2024, March 2024). In this prospective, single-center, cohort study, we enrolled a total of 20 patients who underwent breast reconstruction using the PAP flap between January 2016 and April 2023, consisting of two groups of 10 patients each. Demographic data such as age, body mass index (BMI), comorbidities such as overweight or other severe diseases, complication rate, and smoking habits were collected. This data were analyzed retrospectively. Prospective data were collected at least 12 months postoperatively to evaluate and compare the quality of life using the BREAST-Q questionnaire [21,22] and scar quality of the donor site with the POSAS questionnaire [23], as well as the aesthetic outcome with the Breast Aesthetic scale [24].

Informed consent was signed by all patients for surgery, study participation, publication of data, and photo publication.

Unpaired *t*-tests were used to compare data. Fisher’s exact tests were used to evaluate the differences between the groups. Data compilation and statistical analysis were conducted using ©Microsoft Excel 2016 (Microsoft Corporation; Redmond, WA, USA; https://office.microsoft.com/excel, accessed on 12 November 2019). For all tests, statistical significance was set as a *p*-value less than 0.05.

### 2.2. Patient Data

**Figure 1:**
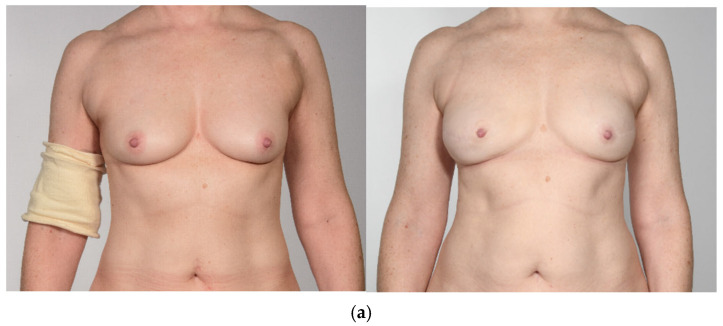

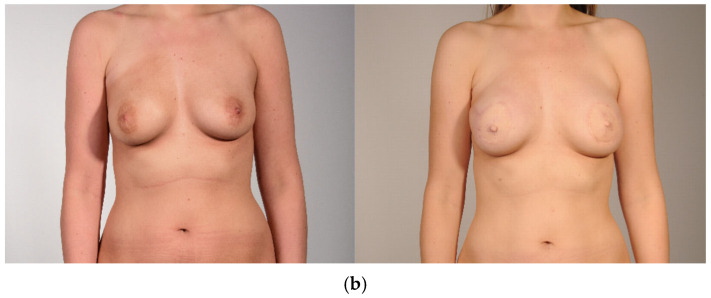
Preoperative (**left**) and postoperative (**right**) view of bilateral breast reconstruction with PAP flap with vertical skin island (**a**) and horizontal skin island (**b**).

Forty-five patients received a PAP flap with a vertical or horizontal skin island design between January 2016 and April 2023 after uni- or bilateral mastectomy. Inclusion criteria were age > 18 years, follow-up time of at least 12 months, diagnosis of breast cancer, high-risk genetic disposition, or recurrent infections of the breast. Exclusion criteria were metastatic disease, Poland’s syndrome, and psychiatric disorders. A total of 30 of the 45 patients received a horizontal skin island design and 15 patients a vertical skin island. We excluded two patients from the vertical group due to metastatic disease and three patients because of the missing preoperative BREAST-Q documentation. In the horizontal skin island group, four patients were excluded because of metastatic disease, two because of psychiatric disorder, three patients because of loss of follow-up, and two patients declined participation. Nine patients with missing preoperative BREAST-Q questionnaires in the horizontal group were also excluded. In all, 20 patients (10 with a vertical skin island design and 10 with a horizontal skin island design) were invited to our Department for clinical examinations, photo documentation, and to complete the postoperative questionnaires. In the vertical skin island group, two patients received adjuvant chemotherapy, and one patient had previous chemotherapy. Four patients underwent adjuvant radiotherapy. In the horizontal skin island group, two patients received adjuvant chemotherapy, and five had previous chemotherapy. Three patients received adjuvant radiotherapy, and three patients had previous radiotherapy. Representative pre- and postoperative images of PAP flap breast reconstruction are shown in Figure 1: (a) vertical design, (b) horizontal design.

### 2.3. Questionnaires

To assess outcomes on quality of life, patients completed the reconstruction module of the BREAST-Q questionnaire version 2.0 in the German language both preoperatively and at least one year postoperatively. Introduced in 2009 by AL Pusic [21], the BREAST-Q is owned by the Memorial Sloan-Kettering Cancer Center and the University of British Columbia. As a validated and standardized questionnaire, it evaluates the patients` satisfaction after breast reconstruction and its impact on psychosocial and sexual health. The questionnaire contains a scale for various themes. Answers vary from “not satisfied” to “very satisfied”. We used an adaptation of the BREAST-Q for the thigh region as a donor site [22] and translated the changed part to German in collaboration with the copyright owners.

Additionally, the Patient and Observer Scar Assessment Scale (The Patient and Observer Scar Assessment Scale, https://www.posas.nl/, accessed on 20 August 2022) [23] was utilized, involving assessments from both patients and surgeons. Both the patient and the observer scales comprise 6 items, each rated on a 10-point scale. A score of “1” indicated normal skin, while a score of “10” signifies the greatest deviation from normal skin. Furthermore, each scale included an overall scar assessment rating.

Standardized photographic documentation of the reconstructed breasts and donor sites was independently evaluated by four plastic surgeons (a female and a male senior plastic surgeon and a male and a female resident). This was conducted by using nine questions of a German translation of the Breast Aesthetic Scale [24]. Each question was rated on a scale from 1 to 5, with 5 indicating the optimal aesthetic outcome. Furthermore, the same plastic surgeons evaluated aesthetic outcomes of the thigh using the questionnaire.

## 3. Results

### 3.1. Patient Characteristics

A total of 20 patients were included in this study. Two groups included 10 patients each. In the vertical skin island group, four patients received bilateral reconstruction with PAP flap (*n* = 14 flaps). Bilateral reconstruction was conducted in five patients (*n* = 15 flaps) in the horizontal skin island group. Evaluation of patient characteristics revealed no significant difference between both groups concerning mean age (vertical skin island group: mean = 44 years, horizontal skin island group: mean = 45 years, *p* = 0.44) and BMI (vertical skin island group: 23.9 kg/m^2^, range: 19.4–32.4 kg/m^2^, horizontal skin island group: 22.7 kg/m^2^, range: 18.5–27.5 kg/m^2^, *p* = 0.35). The reason for reconstruction with PAP flap in the patient with a BMI of 32.4 kg/m^2^ was that she had undergone multiple previous liposuction procedures on the abdomen. Mean flap weight was comparable between groups, with 326.4 g (range: 200–550 g) for the vertical skin island group and 354.7 g (range: 170–550 g) for the horizontal skin island group (*p* = 0.51). Indication for the mastectomy in patients with the vertical skin island design was breast cancer in ten operated breast sites, recurrent infections in two operated breast sites, and prophylactic treatment in two breast sites. One of them had a high genetic disorder. In the horizontal skin island group, the indication for mastectomy was breast cancer in nine operated breasts, recurrent infection in one operated breast, and prophylactic treatment in the remaining breast sites. Three patients had a high-risk genetic disorder. Reconstruction was performed in nine patients as a single-stage procedure in both groups. One patient of each group had autologous reconstruction secondarily to implant-based reconstruction. Detailed patient characteristics are presented in Table 1.

To objectify the complication rates, Clavien–Dindo Classification [25] was used. Complications were categorized into grades 1–3b, with 3b presenting revision surgery under general anesthesia. CD 3a complications were defined as those requiring intervention in local anesthesia. Grade 2 included patients who needed assistance such as aspiration of a hematoma in the ambulance. Patients with other deviations from the normal course were graded 1. In the vertical skin island group, three thighs had a history of complications classified as 3b. The horizontal skin island group included six thighs classified as 3b due to complications such as wound edge necrosis, dehiscence, or hematoma. We further categorized the 3b complications into early complications that occurred immediately postoperatively and required surgical intervention, such as hematoma or infections, and touch-up procedures, where minor revisions were performed during a second hospital stay, such as secondary wound closure. In the horizontal group, four early complications were observed, whereas no early complications occurred in the vertical group (*p* = 0.06, Table 2). Regarding the breast site, five flaps had category 3b in the horizontal skin island group and one in the vertical skin island group (*p* = 0.1, Table 2). A standardized postoperative care protocol was implemented, including regular wound checks and dressing changes during hospitalization, as well as the use of customized compression garments for six weeks postoperatively to promote optimal donor site healing and minimize complications. Detailed outcomes are shown in Table 2.

### 3.2. Results BREAST-Q

All patients completed the BREAST-Q questionnaire before surgery and at least 12 months after surgery. Patients who underwent bilateral reconstruction completed a separate questionnaire for each side.

**Figure 2:**
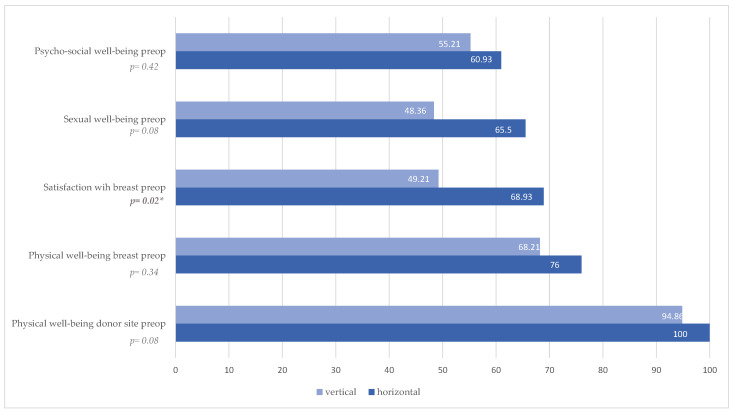
BREAST-Q evaluation preoperative results; * Statistically significant difference (*p* < 0.05).

Evaluation of preoperative BREAST-Q revealed a significantly higher satisfaction with the breast in the horizontal patient group (*p* = 0.02, Figure 2, Table 3). There was no statistically significant difference preoperatively in the other categories, psychosocial well-being (*p* = 0.42, Figure 2, Table 3), sexual well-being (*p* = 0.08, Figure 2, Table 3), and physical well-being breast (*p* = 0.34, Figure 2, Table 3) and physical well-being donor site (*p* = 0.08, Figure 2, Table 3).

**Figure 3:**
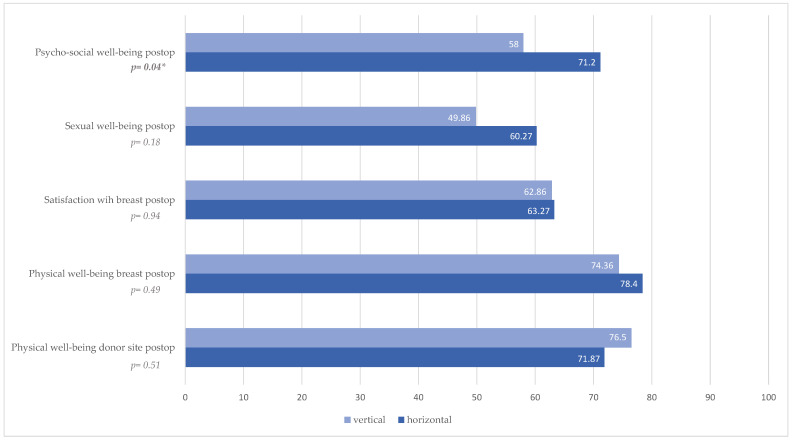
BREAST-Q evaluation postoperative; * Statistically significant difference (*p* < 0.05).

Comparative evaluation of the postoperative BREAST-Q results revealed a significant difference between both groups in the category psychosocial well-being (*p* = 0.04, Figure 3, Table 3). There was no statistical significance in the other categories, sexual well-being (*p* = 0.18, Figure 3, Table 3), satisfaction with breast (*p* = 0.94, Figure 3, Table 3), physical well-being breast (*p* = 0.49, Figure 3, Table 3), and physical well-being donor site (*p* = 0.51, Figure 3, Table 3).

Comparative analysis of the pre- and postoperative results revealed statistically significant differences in the vertical skin island group in the category of satisfaction with breast (*p* = 0.02, Table 4) and in the category of physical well-being of the donor site (*p* = 0.01, Table 4). However, there was no significant difference comparing pre- and postoperative results of the vertical skin island group in the categories of psychosocial well-being (*p* = 0.67), sexual well-being (*p* = 0.08), and physical well-being of the breast (*p* = 0.37). In the horizontal skin island group, there was a significant difference pre- and postoperatively in the category of physical well-being donor site (*p* < 0.01, Table 4). No significant difference comparing pre- and postoperative results of the horizontal skin island group was observed in the categories of psychosocial well-being (*p* = 0.14, Table 4), sexual well-being (*p* = 0.45, Table 4), satisfaction with breast (*p* = 0.47, Table 4), and physical well-being of the breast (*p* = 0.75, Table 4).

**Figure 4:**
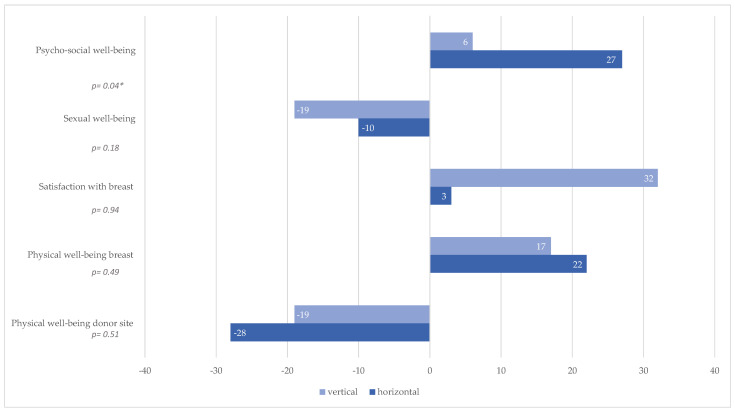
BREAST-Q pre- and postoperative difference %; * Statistically significant difference (*p* < 0.05).

Comparative evaluation of pre- and postoperative outcomes revealed that autologous reconstruction using a PAP flap led to a decrease in sexual well-being and physical well-being of the donor site in both patient cohorts (Table 3 and Table 4, Figure 4).

Additionally, there is a category that specifically focuses on the donor site. In this section, the patients answer three questions about their thigh postoperatively. However, the vertical group shows lower scores across all categories compared to the horizontal group, although these differences did not reach statistical significance (Table 5).

### 3.3. Results POSAS

The POSAS questionnaire was administered to evaluate the scar at the donor site at least 12 months postoperatively. The observer component of the questionnaire was conducted by two plastic surgeons.

Patients’ evaluations of scars showed significant differences, as detailed in Table 6. Those with vertical skin islands perceived their scars as thinner (*p* < 0.01), less irregular (*p* = 0.03), less pigmented (*p* = 0.03), and less stiff (*p* < 0.01).

**Figure 5:**
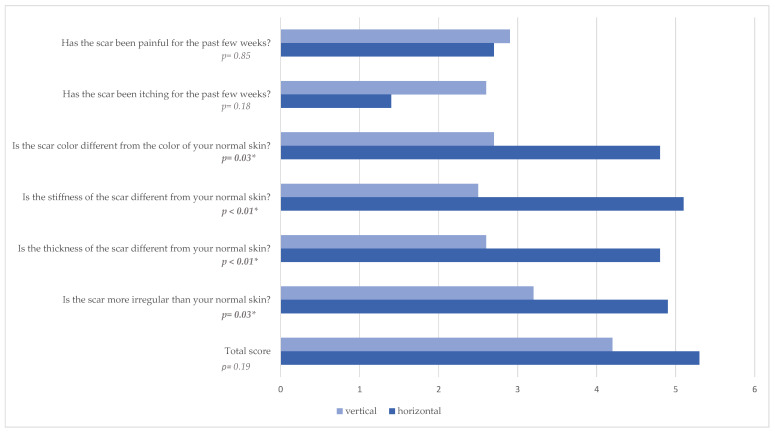
POSAS-score patient scale; * Statistically significant difference (*p* < 0.05).

However, overall assessment of the scar showed no significant difference between both groups (*p* = 0.19, Table 6, Figure 5).

The observers assessed the vertical scar as less pigmented (*p* = 0.03, Table 7), thinner (*p* = 0.02), and with less relief (*p* = 0.02). The total score was given as 2.7 in the vertical group and as 3.2 in the horizontal group (*p* = 0.46).

### 3.4. Cosmetic Results

For the cosmetic evaluation of the donor site, a modified version of the Breast Aesthetic scale [25] was used. Items included thigh symmetry, position of the gluteal fold, shape and contour, scar appearance, and overall evaluation of the donor site. In the Breast Aesthetic Scale, the scoring goes from 1 to 5, whereas 1 indicates a “very poor” result and 5 indicates a “very good” result. We used the same scoring for the questionnaire of the donor site.

**Figure 6:**
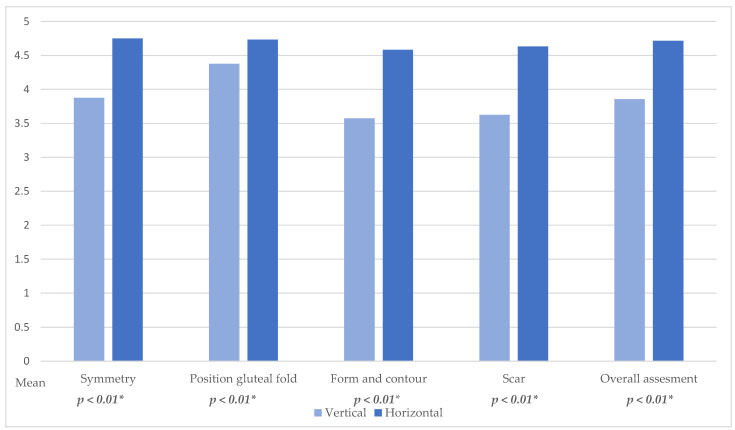
Thigh aesthetic evaluation; * Statistically significant difference (*p* < 0.05).

Analysis of aesthetic outcome of the thigh revealed a significantly better cosmetic result in horizontal skin island group throughout all categories, symmetry of the thigh (*p* < 0.01, Figure 6), position of the gluteal fold (*p* < 0.01, Figure 6), form and contour of the thigh (*p* < 0.01, Figure 6), appearance of the scar (*p* < 0.01, Figure 6), and overall appearance (*p* < 0.01, Figure 6).

Notably, in all categories, the horizontal skin paddle donor site received higher scores from all four evaluators. Most categories were significantly higher rated by at least three of the four evaluators. Analysis of symmetry (*p* < 0.01, Figure 6) and scar appearance (*p* < 0.01, Figure 6) showed significantly better scores in the horizontal skin island group in every evaluation by the surgeons.

**Figure 7:**
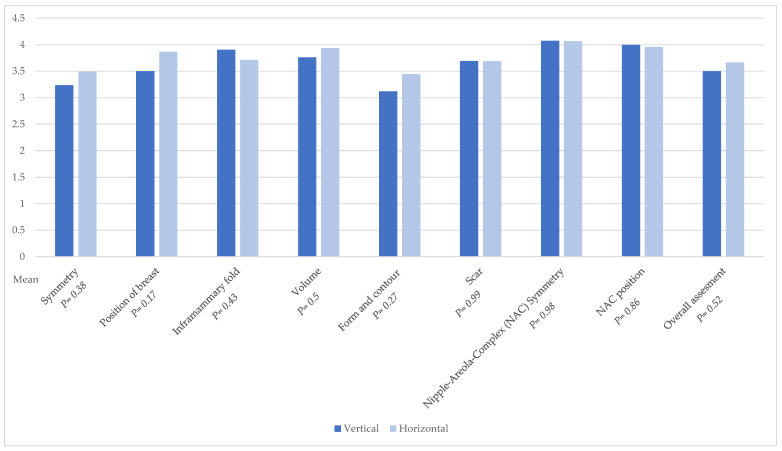
Breast aesthetic evaluation.

**Figure 8:**
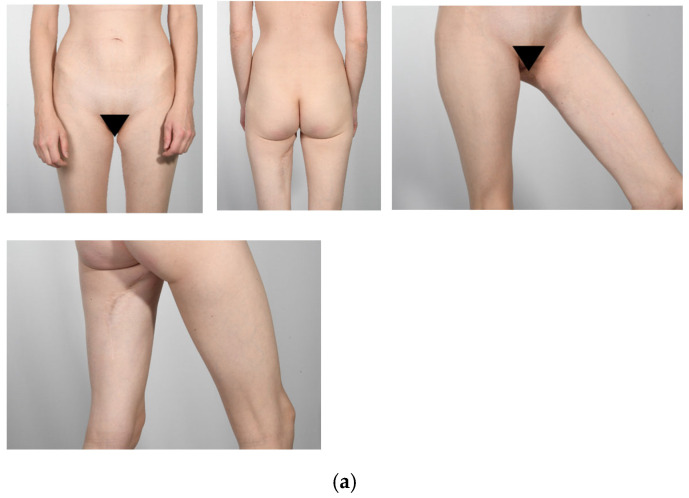

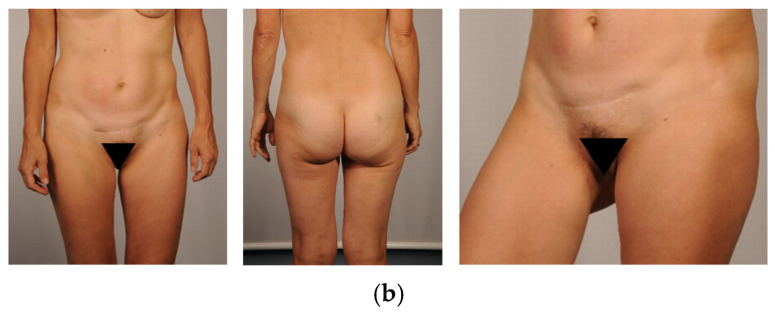
Postoperative view of donor sites of unilateral breast reconstruction with PAP flap with vertical skin island (**a**) and horizontal skin island (**b**).

Assessing cosmetic results of the breast, we used the Breast Aesthetic Scale [25]. Four gender-balanced plastic surgeons, consisting of two residents and two board-certified senior surgeons, analyzed the donor site and the aesthetic outcome of the breast. The questionnaire was completed based on standardized postoperative photo documentation. A comparative evaluation of both patient groups revealed no significant differences in the aesthetic outcome of the breast (Figure 7). Pre- and postoperative images of the donor site are presented in Figure 8.

## 4. Discussion

In recent years, the PAP flap has emerged as a suitable alternative to the DIEP flap. Initially utilized when the DIEP flap is not feasible, recent studies [6,8,9,11,15] have shown comparable aesthetic and complication rate-related data. Thus, the PAP flap is not just a second-line alternative for patients with limited donor tissue or limiting scars due to prior abdominal surgeries, but a potential option for all patients favoring autologous tissue breast reconstruction. The thigh-based PAP flap is characterized by a long pedicle, continuous blood supply, and favorable scar placement [26]. At our department, the PAP flap is mainly used in slim patients lacking sufficient abdominal donor tissue. While this subgroup is a frequent indication in our practice, we acknowledge that the PAP flap is also broadly used in patients with adequate abdominal tissue, particularly in cases requiring low-volume reconstruction or when abdominal donor site morbidity is to be avoided. The focus on this subgroup in our study reflects institutional case distribution rather than a general limitation of the PAP flap’s applicability. Originally, we started with the classic horizontal design. However, this presented a major challenge in harvesting enough tissue for lean patients. In such cases, obtaining sufficient volume while preserving optimal contour is technically demanding, requiring precise dissection and careful flap shaping. An additional challenge in slim patients is that the PAP flap is already associated with a higher donor site complication rate [15]. This risk is further exacerbated by the need to harvest as much tissue as possible, potentially leading to an increased incidence of wound healing issues and contour deformities. To address this, both technical refinements and alternative flap designs have been explored [7,14,19,20]. Alternative flap designs have proven effective in obtaining more reconstructive tissue and thus offering enough tissue for higher reconstruction volume [16,27,28,29]. Our department observed a higher rate of wound healing complications up to 29.6% using the PAP flap with the original horizontal skin island design [11,14]. This is likely because our PAP flap patients are challenging to treat due to their low BMI. Therefore, we first refined the technique [14] and then planned the PAP flap as a vertical flap inspired by the work of Rivera-Serrano et al. [18] and Scagliogni et al. [19]. The vertical skin island design was introduced at our department by one of the senior authors as part of an institutional effort to optimize donor-site outcomes. While she led the initial application of the vertical design, the procedures were performed as part of the breast reconstruction team and not by a single surgeon in isolation. In bilateral cases, flap harvest was shared with a second surgeon, and the vertical design was gradually taught and implemented beyond the initial surgeon. Therefore, while early vertical cases were predominantly performed by one senior surgeon, the technique’s adoption reflects a broader institutional evaluation rather than individual advocacy. In this study, we present our data on the vertical skin island in comparison to the horizontal skin island and the influence of skin island design on complication rates, patients’ satisfaction, and aesthetic outcome. The two patient groups were matched in terms of age, BMI, mastectomy volume, and flap volume. Also, there is no statistically significant difference concerning chemotherapy, radiotherapy, and smoking status. Although we did not observe a statistically significant difference in smoking status, smoking may still affect wound healing.

We acknowledge that the limited sample size of this study reduces the statistical power to draw definitive conclusions regarding complication rates or long-term aesthetic outcomes. While larger series have been published focusing predominantly on the horizontal PAP flap, direct comparisons between vertical and horizontal skin paddle designs remain rare. To our knowledge, only two published studies to date have investigated this specific comparison with larger patient numbers [17,28], and other reports on the vertical PAP flap involve similar or smaller cohorts [18,19]. Several large case series have further contributed valuable data on the outcomes of horizontal PAP flap breast reconstruction. Allen et al. reported excellent results with 164 flaps and minimal flap loss [30]. Haddock and colleagues subsequently presented two extensive series of 101 and 265 flaps, demonstrating low flap loss rates and progressive refinements to reduce complications [7,31]. More recently, Tielemans et al. published outcomes of an extended PAP flap design in 46 cases to increase flap volume for larger reconstructions or patients with limited donor tissue [27]. In this context, our study offers one of the few matched cohort comparisons of vertical versus horizontal designs, providing additional insights into donor site outcomes, patient-reported outcomes (PROMs), and early postoperative complications. We recognize that further prospective studies with larger sample sizes will be necessary to confirm and expand upon these preliminary findings.

In our findings, there were reduced rates of early complications, particularly wound healing, at the donor site when using the vertical skin island design. Although not statistically significant, we noticed this as a positive trend. Scaglioni et al. [19] and Artz et al. [20] have reported similar findings in previous studies. We believe that with the vertical skin island, less tension is placed on the scar. At the horizontal incision line, the scar is placed directly in an area with high tension, which tends to result in wound-healing complications. However, the early complication rate in the horizontal group was significantly higher than in the vertical group (*p* = 0.06). Our findings suggest that a vertical skin island design may be particularly beneficial in low BMI patients by reducing complication rates.

While the vertical group showed more touch-up procedures, the horizontal group experienced a higher rate of early complications requiring immediate revision. One possible explanation is that the vertical scar does not lie within a natural anatomical fold, which may lead to increased scar visibility or irritation during healing, making it more likely to require secondary revision. In contrast, the horizontal scar is positioned within a natural gluteal crease, which may promote better scar integration and tolerance, even if early complications are more frequent. Therefore, the clinical relevance of each complication type must be considered in context.

In the postoperative BREAST-Q questionnaire, no significant difference is observed between the two groups (vertical skin island versus horizontal skin island) in all categories except the psychosocial well-being, which is described significantly better in the horizontal group (*p* = 0.04). A possible explanation for this could be that the patients with vertical skin island design were mainly operated on during the COVID-19 pandemic, which had a negative impact on their psychosocial well-being in general. The horizontal skin island group underwent surgery mainly before the pandemic. However, we noted that both groups were significantly less satisfied with their thigh postoperatively compared to the preoperative results (*p* = 0.01 in the vertical skin island group versus *p* < 0.01 in the horizontal skin island group). Although the differences in BREAST-Q scores related to the thigh were not statistically significant, we observed a trend toward lower patient-reported satisfaction in the vertical group across several domains, including the appearance of the thigh when unclothed, the position of the gluteal crease, and scar-related concerns. These findings suggest that, despite potential advantages in scar quality or donor-site tension with the vertical design, the overall perception of thigh aesthetics may be less favorable. This highlights the importance of discussing aesthetic advantages and disadvantages with patients during preoperative counseling, particularly when choosing between skin island designs.

Evaluating the POSAS questionnaire, significantly greater satisfaction was noted both among the observers and the patients in the vertical skin island group. The scar was described as thinner (*p* < 0.01), less irregular (*p* = 0.03), less pigmented (*p* = 0.03), and less stiff (*p* < 0.01) from the patients and as less pigmented (*p* = 0.03), thinner (*p* = 0.02), and with less relief (*p* = 0.02) from the observers. We attribute this to the location of the scar, as with the vertical dorsal design, patients themselves see their scars poorly. The horizontal scar is much more visible and bothersome to patients, especially when sitting. Further, we think this result is caused by reduced rates of wound healing complications. Another advantage of the vertical skin island design is a more accessible surgical site. With the vertical skin island design, access to the pedicle is easier, as it eliminates the need to undermine the flap up to the distal thigh, compared to the horizontal skin island. Furthermore, advantages of the vertical skin island design over the horizontal design include reduced rate of wound healing complications at donor site, better access to the pedicle intraoperatively, fewer lymphatic vessel injuries [19], prevention of widening of the labia majora [32], and better quality of life for patients according to BREAST-Q. We did not observe that the alternative flap design resulted in a higher flap weight, nor was it our aim to obtain larger flaps with comparable patient demographics.

In our aesthetic analysis of the donor site, we found that the horizontal skin island design was superior to the vertical skin paddle in all categories. Particularly in terms of symmetry, the horizontal skin island design showed better results. Initially, we were concerned that the horizontal skin paddle would perform worse regarding symmetry and position of the gluteal fold. However, this concern was not confirmed. Although statistical significance was achieved by only two out of four evaluators for the position of the gluteal fold, a trend is evident. Surprisingly, thigh symmetry was rated better by all four surgeons for the horizontal skin paddle. This may be due to the vertical skin paddle leaving more contour defects than anticipated. The overall assessment of the appearance of the scar from the horizontal skin paddle was rated as superior in aesthetic evaluation by all surgeons, with three out of four showing statistically significant results. Interestingly, although patient-rated POSAS scores indicated better scar characteristics in the vertical group, such as thinner, less pigmented, and more flexible scars, the standardized aesthetic evaluations based on postoperative photographs evaluated by surgeons consistently favored the horizontal design across all measured thigh parameters. This highlights an important point: favorable scar quality alone does not necessarily translate into superior overall aesthetic outcomes. Factors such as scar placement, symmetry, and how well the contour integrates into natural anatomical landmarks appear to significantly influence the aesthetic appearance. These findings demonstrate the need to consider both subjective and objective outcome measures when evaluating donor site design.

Also, our concern that the vertical design would lead to a more vertically shaped breast was not confirmed. The aesthetic analysis of breasts showed no statistical significance in all categories. It can be concluded that the different skin island designs do not affect breast shape.

Moreover, while our study emphasizes early postoperative outcomes and patient-reported aesthetics, we recognize the importance of long-term follow-up. Future analyses will aim to include outcomes such as sensory changes, donor site morbidity, and the need for secondary revision surgery. Continued follow-up of this cohort is ongoing, and we believe that prospective, larger-scale studies will be essential to further validate and refine our findings.

This study shows the discrepancy between patients’ versus surgeons’ evaluations concerning aesthetic aspects. Therefore, we recommend implementing PROMs in general regarding aesthetic aspects as standardized quality indicators.

As a limitation, we would like to address the single-institution design, which provides insight only into the outcomes from our department. Another limitation is the non-randomized study design. While the horizontal skin island procedures were performed by multiple senior surgeons, the vertical skin island technique was primarily introduced and performed by one senior surgeon during its early implementation phase. However, in bilateral cases, one side was typically performed by a second surgeon, and the vertical design was gradually adopted and taught to other members of the breast reconstruction team. Although this surgeon was also involved in horizontal flap procedures, surgeon-related variability in outcomes cannot be entirely ruled out. Another limitation could be the sample size of the patients; however, a key strength of the study lies in the matching of patients, which ensures homogeneity between the two groups included.

## 5. Conclusions

The results of our study emphasize the advantages of employing a vertical skin island in PAP flap breast reconstruction. This modified design appears to offer notable benefits in terms of complication rate, wound healing, and scar management. The reduced need for thigh undermining, coupled with improved aesthetic satisfaction, positions the vertical skin island design as a viable and potentially superior option for breast reconstruction compared to the traditional horizontal skin island approach. The vertical skin island design presents a promising approach to handle donor site complication rates in challenging breast reconstruction with PAP flap in low BMI patients. These findings suggest that vertical scars may offer technical and healing-related benefits. However, the horizontal design appears to be preferred from an aesthetic aspect, due to better contour and gluteal symmetry. This highlights the need for individualized preoperative counseling that addresses both scar quality and aesthetic priorities. Although the vertical flap design achieved significantly poorer results in the aesthetic evaluation by surgeons, this must be weighed against lower complication rates and better scar quality outcomes. Our findings contribute to the ongoing evolution of surgical techniques in the field of breast reconstruction, aiming to optimize the overall experience and outcomes for breast cancer patients.

## Figures and Tables

**Table 1:** Demographics.

	Vertical (n * = 10, ‘n = 14)		Horizontal (n * = 10, ‘n = 15)		
	MEAN	SD	MEAN	SD	*p* *
Age (years)	43.8	9.8	44.6	6.5	0.44
BMI	23.9	4.4	22.7	2.7	0.35
Flap volume (cc)	326.4	86.1	354.7	133.5	0.51
Mastectomy volume (cc)	343.1	103.9	324.7	153	0.71
	n	(%)	n	(%)	
**Time of reconstruction ‘**					
Primary	12	85.7	13	86.7	
Secondary	2	14.3	2	13.3	
Radiotherapy *	4	40	6	60	0.33
Adjuvant	4	40	3	30	
Previous radiotherapy	0	0	3	30	
Chemotherapy *	3	30	7	70	0.09
Adjuvant	2	20	2	20	
Previous chemotherapy	1	10	5	50	
Positive genetic testing *	1	10	3	30	
Active smoker *	4	40	1	10	0.98
**Indication for mastectomy ‘**					
Breast cancer	10	71.4	9	60	0.70
Prophylactic	2	14.3	5	33.3	0.17
Others	2	14.3	1	6.7	

* n = patients, ‘n = flaps; * Statistically significant difference (*p* < 0.05).

**Table 2:** Postoperative complications.

	Vertical (‘n = 14)	%	Horizontal (‘n = 15)	%	*p* *
Thigh					
1	2	14.3	3	20	0.53
2	0	0	0	0	
3a	1	7.1	0	0	0.48
3b (in total)	3	21.4	6	40	0.25
3b: early complications	0	0	4	26.7	0.06
3b: touch-up procedures	3	21.4	2	13.3	0.86
Breast					
3b	1	7.1	5	33.3	0.1

‘n = flaps; * Statistically significant difference (*p* < 0.05).

**Table 3:** BREAST-Q.

	Vertical (‘n = 14)		Horizontal (‘n = 15)		
	MEAN	SD	MEAN	SD	*p* *
Psychosocial well-being preop	55.2	15.9	60.9	21.7	0.42
Psychosocial well-being postop	58	18.5	71.2	13.9	0.04 *
Percentage difference preop to postop	6	17	27	39	0.08
Sexual well-being preop	48.4	22.1	65.5	20.1	0.08
Sexual well-being postop	49.9	25.1	60.3	12.8	0.18
Percentage difference preop to postop	3	19	−10	24	0.2 *
Satisfaction with breast preop	49.2	15.4	68.9	26.4	0.02 *
Satisfaction with breast postop	62.9	13.4	63.3	13.5	0.94
Percentage difference preop to postop	32	21	3	38	0.02 *
Physical well-being breast preop	68.1	19.2	76	23.8	0.34
Physical well-being breast postop	74.4	16	78.4	15.3	0.49
Percentage difference preop to postop	17	44	22	76	0.80
Physical well-being donor site preop	94.9	10.2	100	0	0.08
Physical well-being donor site postop	76.5	19.6	71.9	17.8	0.51
Percentage difference preop to postop	−19	21	−28	19	0.23

‘n = flaps; * Statistically significant difference (*p* < 0.05).

**Table 4:** BREAST-Q difference pre- and postoperative; * Statistically significant difference (*p* < 0.05).

	*p* *
**Psychosocial well-being**	
Difference pre- and postoperative vertical	0.67
Difference pre- and postoperative horizontal	0.14
**Sexual well-being**	
Difference pre- and postoperative vertical	0.08
Difference pre- and postoperative horizontal	0.45
**Satisfaction with breast**	
Difference pre- and postoperative vertical	0.02 *
Difference pre- and postoperative horizontal	0.47
**Physical well-being breast**	
Difference pre- and postoperative vertical	0.37
Difference pre- and postoperative horizontal	0.75
**Physical well-being donor site**	
Difference pre- and postoperative vertical	0.01 *
Difference pre- and postoperative horizontal	<0.01 *

**Table 5:** BREAST-Q thigh.

	Vertical (‘n = 14)		Horizontal (‘n = 15)		
	MEAN	SD	MEAN	SD	*p* *
How does the thigh look unclothed	2.5	1.1	3	0.8	0.14
Position horizontal gluteal crease	2.8	1.3	3	0.6	0.25
Scar	2.5	1.1	3	0.9	0.17

‘n = flaps; * Statistically significant difference (*p* < 0.05).

**Table 6:** POSAS-score patient scale.

	Vertical (‘n = 14)		Horizontal (‘n = 15)		
	MEAN	SD	MEAN	SD	*p* *
Has the scar been painful for the past few weeks?	2.9	2.9	2.7	2.4	0.85
Has the scar been itching for the past few weeks?	2.6	2.9	1.4	1.1	0.18
Is the scar color different from the color of your normal skin?	2.7	2.5	4.8	2.4	0.03 *
Is the stiffness of the scar different from your normal skin?	2.5	1.7	5.1	2.4	<0.01 *
Is the thickness of the scar different from your normal skin?	2.6	1.9	4.8	2.2	<0.01 *
Is the scar more irregular than your normal skin?	3.2	1.8	4.9	2	0.03 *
Total score	4.2	3	5.3	2.2	0.19

‘n = flaps; * Statistically significant difference (*p* < 0.05).

**Table 7:** POSAS-score observer scale.

	Vertical (‘n = 14)		Horizontal (‘n = 15)		
	MEAN	SD	MEAN	SD	*p* *
Vascularity	2.6	1.6	2.5	1	0.45
Pigmentation	2.6	1.3	4	1.7	0.03 *
Thickness	2.3	1.1	3.3	1	0.02 *
Relief	2.4	0.9	3.6	1.5	0.02 *
Pliability	1.9	1.4	2.3	0.8	0.31
Surface area	3.6	1.4	3.4	1.7	0.8
Total score	2.7	1.5	3.2	0.2	0.46

‘n = flaps; * Statistically significant difference (*p* < 0.05).

## Data Availability

The data presented in this study consist of anonymized patient information and are available from the corresponding author upon reasonable request. Public access is restricted due to ethical considerations and institutional data protection policies.

## Abbreviations

The following abbreviations are used in this manuscript:

PAP flap	Profunda artery perforator flap
DIEP flap	Deep inferior epigastric artery flap
NAC	Nipple areola complex
PROMs	Patient-reported outcome measures
